# Serum estradiol should be monitored not only during the peri-menopausal period but also the post-menopausal period at the time of aromatase inhibitor administration

**DOI:** 10.1186/1477-7819-7-88

**Published:** 2009-11-12

**Authors:** Taeko Nagao, Misako Kira, Masako Takahashi, Junko Honda, Toshiyuki Hirose, Akira Tangoku, Hitoshi Zembutsu, Yusuke Nakamura, Mitsunori Sasa

**Affiliations:** 1 Department of Oncological and Regenerative Surgery, Institute of Health Biosciences, The University of Tokushima, 3-18-15, Kuramoto-Cho, Tokushima 770-8509, Japan; 2 Department of Surgery, Tokushima Breast Care Clinic, 4-7-7, Nakashimada-Cho, Tokushima 770-0052, Japan; 3 Department of Surgery, National Higashi Tokushima Hospital, 1-1, Ohmukai-kita, Ootera, Itano, Tokushima 779-0193, Japan; 4 Human Genome Center, Institute of Medical Science, The University of Tokyo, 4-6-1, Shirokanedai, Minato-Ku, Tokyo 108-8639, Japan

## Abstract

**Background:**

Aromatase inhibitor (AI) therapy is being extensively used as postoperative adjuvant therapy in patients with hormone receptor-positive postmenopausal breast cancer. On the other hand, it has been reported that ovarian function was restored when AI was administered to patients who had undergone chemical menopause with chemotherapy or tamoxifen. However, there have been no reports of comprehensive monitoring of estradiol (E2) in breast cancer patients with ordinary menopause who were being administered AI.

**Patients and Methods:**

Beginning in March 2008, regular monitoring of the serum levels of E2, luteinizing hormone (LH) and follicle-stimulating hormone (FSH) was performed for 66 postmenopausal breast cancer patients who had been started on AI therapy. For this study, we chose anastrozole as the AI. The assays of those hormones were outsourced to a commercial clinical laboratory.

**Results:**

In 4 of the 66 patients the serum E2 level was decreased at 3 months but had then increased at 6 months, while in 2 other patients E2 was decreased at both 3 and 6 months but had increased at 9 months.

**Conclusion:**

The results indicate that, in some breast cancer patients with ordinary menopause, E2 rebounds following AI therapy. In the future, E2 monitoring should be performed for a larger number of patients being administered AI therapy.

**Trial registration:**

Our trial registration number is 19-11-1211.

## Introduction

Approximately 60~70% of breast cancers are hormone receptor-positive, and tamoxifen (TAM) has been extensively used as postoperative adjuvant therapy. More recently, aromatase inhibitors (AIs) have been developed, and they have greatly altered the therapeutic strategy for hormone receptor-positive postmenopausal breast cancer [1,2]. The results of large-scale clinical studies have demonstrated that AI therapy is more useful than TAM, and today AIs are widely employed as standard therapy such as by switching from TAM to an AI, performance of extended AI therapy after 5-year administration of TAM, and as a first-line agent as postoperative adjuvant therapy [3-8]. On the other hand, it has been reported that ovarian function was restored in some patients when AI was administered to patients who had undergone chemical menopause by means of chemotherapy or TAM [9-11]. AI expresses its antitumor effect by reducing the serum level of estradiol (E2), and clinical efficacy of AI cannot be expected in patients in whom the E2 level does not decrease in response to AI. Such special patients have been reported, but there have been no reports of E2 monitoring in patients who underwent ordinary menopause and were administered AI as adjuvant therapy for breast cancer. Accordingly, beginning in March 2008, we performed regular monitoring of the serum level of E2, luteinizing hormone (LH) and follicle-stimulating hormone (FSH) in postmenopausal breast cancer patients who were administered an AI.

## Patients and Methods

The patients included in this study were 66 patients who had been started on AI therapy between March 2008 and February 2009 at Tokushima Breast Care Clinic and underwent regular monitoring of their clinical course and for whom detailed clinicopathological studies were possible. In this study, we administered only anastrozole as an AI. The patient compliance in ingesting the AI was verbally confirmed, and patients with poor compliance were excluded from this study. Each patient was administrated anastrozole 1 mg/body p.o. once daily. Testing and evaluation of estrogen receptor (ER), progesterone receptor (PgR) and human epithelial growth factor receptor type 2 (HER2; Hercep Test, Dako) were performed by the conventional immunohistochemical methods [12]. At Tokushima Breast Care Clinic, the criterion for indication of AI administration is hormone receptor-positive, postmenopausal breast cancer. Menopause was defined as the state of having undergone bilateral oophorectomy, or women aged 60 years or older, aged under 60 years with amenorrhea for at least 12 months, and serum E2, LH and FSH levels satisfying the diagnostic criteria for postmenopause. The 66 patients entered in the present study consisted of 65 women who received the AI as postoperative adjuvant therapy and one woman who received the AI as preoperative therapy. The clinical disease stage showed a range of I~IV, and the surgical procedures consisted of mastectomy in 12 patients, breast-conserving surgery in 53 patients and only core needle biopsy in one patient. Prior to administration of the AI, 12 patients had been administered TAM, 9 patients had undergone chemotherapy, and 5 patients had received both chemotherapy and TAM. The remaining 40 patients were treated only with the AI (Additional File 1).

The serum levels of E2, LH and FSH were assayed prior to administration of the AI and then at 3, 6, 9 and 12 months after starting the AI therapy. Those assays were performed by FALCO Biosystems Ltd. (Kyoto, JAPAN), and serum E2, LH and FSH levels were measured by ECLIA (Electrochemiluminescence Immunoassay) in this laboratory. The laboratory's standard values for the postmenopausal levels of those hormones are 10-40 pg/mL for E2 (lower limit of detection: 5 pg/mL), 7.7-58.5 mIU/mL for LH and 25.8-134.8 mIU/mL for FSH.

Statistical analyses were performed using the chi-square test and the Wilcoxon signed ranks test, and a p value of < 0.05 was defined as indicating significance.

The design of this study was approved by the Ethics Committee of The Institute of Medical Science, The University of Tokyo, and The University of Tokushima. Prior informed consent was obtained in writing from each of the enrolled patients.

## Results

**Figure 1:**
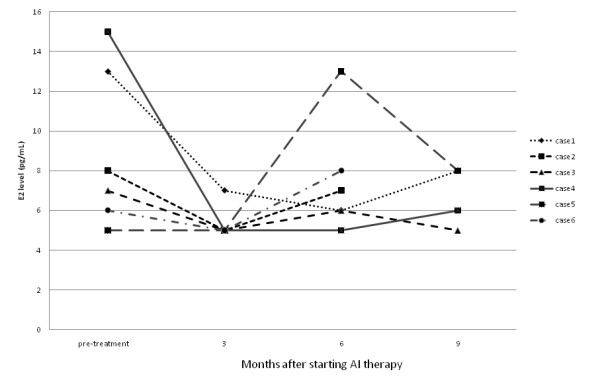
**Serum E2 level in cases with E2 rebounded**. In 4 patients (Nos. 2, 3, 5, 6), the E2 level had increased at 6 months after starting AI (anastrozole) therapy, and in 2 patients (Nos. 1, 4), the E2 level had increased at 9 months after starting AI therapy.

The mean observation time for the patients was 5.9 months. Additional File 2 shows the data for the serum levels of E2, LH and FSH for all of the patients. Because the lower limit of detection for E2 was 5 pg/mL, values of 5 pg/mL or less were recorded as 5 pg/mL. The baseline value of E2 (i.e., prior to AI administration) was already at the lower limit of detection (5 pg/mL) in 24 (36%) of the patients. The mean E2 level was significantly reduced at both 3 months (5.3 ± 0.8 pg/mL) and 6 months (5.7 ± 1.7 pg/mL) after the start of AI administration compared with the mean baseline value (7.8 ± 3.3 pg/mL). However, in 4 patients the E2 level had increased again at 6 months even though it had been clearly decreased at 3 months after the start of AI administration, while in 2 other patients the E2 level had increased again at 9 months in spite of having been decreased through 6 months after starting AI administration (Fig. 1). In addition, the assay results also showed that, due to the administration of the AI, the mean serum level of FSH was significantly increased from the baseline value of 55.3 ± 31.3 pg/mL to 65.3 ± 32.6 pg/mL after 3 months and 65.4 ± 35.4 pg/mL after 6 months. The mean serum level of LH was also observed to increase with time following the start of AI administration, but there was no clearly significant difference compared with the baseline (Additional File 2). In 5 of these 6 patients with E2 rebound, the FSH level was increased at 3 months after the start of AI administration, while in one patient FSH was not changed. In addition, the LH level was increased in 2 of those patients, unchanged in 2 patients and decreased in the remaining 2 patients.

Additional File 1 presents a comparison of the clinicopathological findings for the patients whose serum E2 was continuously decreased after the start of AI administration and the patients whose E2 level later rebounded. The clinical disease stage was 0 or I in each of the patients whose E2 rebounded. The E2 showed a continuous decrease in 60 of the patients, 25 (41.7%) of whom had undergone some sort of therapy prior to AI administration, whereas only one (16.7%) of the patients with E2 rebound had undergone prior therapy. Next, we examined for correlations between the serum E2 level and the age at menarche, the number of years since menopause, the number of births and the body mass index (BMI) of the patients. The mean age at menarche was 15.5 ± 1.2 years in the patients with E2 rebound, which was significantly higher than the mean age of 13.7 ± 1.6 years in the patients with continuous E2 reduction. Moreover, although the difference did not reach statistical significance, the number of births experienced by the women showing E2 rebound was smaller (Additional File 3).

## Discussion

The advent of AIs marked a revolution in the therapeutic approach to postmenopausal, hormone receptor-positive breast cancer [1,2]. AIs include three third-generation compounds, anastrozole, exemestane and letrozole, and large-scale clinical trials have been carried out on each [3-8]. Based on the results of those trials, AIs have come to be extensively used to treat hormone receptor-positive, postmenopausal breast cancer. AI therapy brings about an approximately 90% reduction in the serum and tumor-tissue levels of E2 in postmenopausal women, which is the basis of the expression of the antitumor effects of AIs on hormone receptor-positive breast cancer cells [13-17]. On the other hand, there have been reports that AI administration led to restoration of the ovarian function in some patients with chemically induced menopause or whose ovarian function was being suppressed by administration of TAM [9-11]. Those can be thought to be special cases in which menstruation was restarted due to AI administration after chemical menopause had been induced in premenopausal patients by chemotherapy or TAM administration. However, there have been no reports of comprehensive monitoring of E2 in breast cancer patients who had undergone ordinary menopause and were being administered an AI. For that reason, we initiated the present clinical study in March 2008 to monitor and analyze the serum levels of E2, LH and FSH in postmenopausal breast cancer patients who were started on AI (anastrozole) therapy. The assays of those serum hormone levels were subcontracted to an outside, government-licensed commercial clinical laboratory. The assay results indicated the possibility that the serum E2 level rebounds in some postmenopausal women due to administration of an AI. The E2 level shows circular rhythm, but we did not investigate that in this study. Moreover, intra-and inter-assay error values may occur with this ECLIA assay. We also did not investigate the precision of the ECLIA method. However, it is at least clear that in these patients the reduction of E2 that can be expected due to the effects of AI therapy is not being achieved. As one potential reason for this phenomenon, we surmised the possibility that there is some residual ovarian function in those patients even though they were thought to be clinically postmenopausal, and that a negative feedback due to the E2 reduction induced by administration of an AI leads to E2 secretion from the residual ovarian tissue in response to stimulation with LH and FSH. Almost all of the patients in this study who showed renewed elevation of serum E2 had not undergone any prior therapy and were administered only the AI, and the results indicate the possibility that they possessed residual ovarian function in spite of being approximately 60 years of age. Therefore, defining "postmenopausal" only on the basis of an age of "60 years" may be erroneous. Moreover, it was indicated that there is a possibility of greater residual ovarian function in patients in whom the age of menarche is delayed. On the other hand, it can be surmised that administration of chemotherapy or TAM will further suppress any residual ovarian function in women who would ordinarily be assumed to be postmenopausal on the basis of their age, and that, due to the complete absence of residual ovarian function, administration of an AI would result in continuous inhibition of serum E2. The data generated in the clinical studies of AIs did not show any patients in whom the AI administration led to renewed elevation of E2 [13-16]. We assume that the reason for this is that most of the patients enrolled in those clinical trials had advanced, postmenopausal, recurrent breast cancer and that almost all had undergone prior treatment using chemotherapy and/or TAM. Moreover, it can be thought that there is a possibility that the inhibition of serum E2 was continuous in all of those study patients. Also, in women who had undergone pretreatment with chemotherapy and/or TAM prior to menopause, a reviced definition may be necessary for "postmenopausal". As another potential reason, we postulate the possibility that, in some patients, the expression of the effects of AI therapy is inadequate due to a genetic aberration in relation to an AI-metabolizing enzyme such as CYP19 (aromatase P450) [18,19]. We hope that further studies will be carried out to clarify this issue.

For this study, the lower limit of detection for serum E2 was 5 pg/mL. Based on the study results, it can be concluded that the serum E2 level was 5 pg/mL or less even before AI administration in approximately 36% of the patients who were judged to be clinically postmenopausal. That incidence needs to be further investigated by means of a more sensitive assay method. In such patients, any further decrease in serum E2 cannot be detected.

The limitations of this study are the small number of enrolled patients and the rather short follow-up time of approximately one-half year. In addition, as an AI, only anastrozole was administered and investigated. Nevertheless, we surmise that, since AIs are poised to play a key role in the treatment of postmenopausal hormone receptor-positive breast cancer patients, it is necessary to carry out further investigation of the effects of AI therapy on the serum E2 level by performing E2 monitoring in a much larger number of patients.

## Abbreviations

AI: aromatase inhibitor; E2: estradiol; LH: luteinizing hormone; FSH: follicle-stimulating hormone; TAM: tamoxifen; ER: estrogen receptor; PgR: progesterone receptor; HER2: human epithelial growth factor receptor type 2; ECLIA: Electrochemiluminescence immunoassay

## Competing interests

The authors declare that they have no competing interests.

## Authors' contributions

MS initiated and co-wrote the paper with TN, MK and AT. MT, JH, and HT took part in the care of patients. ZH and YN helped in preparation of the manuscript. All authors read and approved the manuscript.

## References

[B1] Goldhirsch A Wood W Gelber R Coates A Thürlimann B Senn H Panel Members Progress and promise: highlights of the international expert consensus on the primary therapy of early breast cancer 2007 Ann Oncol 2007 18 1133 1144 10.1093/annonc/mdm271 17675394

[B2] Winer EP Hudis C Burstein HJ Wolff AC Pritchard KI Ingle JN Chlebowski RT Gelber R Edge SB Gralow J Cobleigh MA Mamounas EP Goldstein LJ Whelan TJ Powles TJ Bryant J Perkins C Perotti J Braun S Langer AS Browman GP Somerfield MR American society of clinical oncology technology assessment on the use of aromatase inhibitors as adjuvant therapy for postmenopausal women with hormone receptor-positive breast cancer: Status report 2004 J Clin Oncol 2005 23 619 629 10.1200/JCO.2005.09.121 15545664

[B3] The Arimidex, Tamoxifen, Alone or in Combination (ATAC) Trialists' Group Effect of anastrozole and tamoxifen as adjuvant treatment for early-stage breast cancer: 100-month analysis of the ATAC trial Lancet Oncol 2008 9 45 53 10.1016/S1470-2045(07)70385-6 18083636

[B4] Coates AS Keshaviah A Thürlimann B Mouridsen H Mauriac L Forbes JF Paridaens R Castiglione-Gertsch M Gelber RD Colleoni M Láng I Mastro LD Smith I Chirgwin J Nogaret J Pienkowski T Wardley A Jakobsen EH Price KN Goldhirsch A Five years of letrozole compared with tamoxifen as initial adjuvant therapy for postmenopausal women with endocrine-responsive early breast cancer: Update of study BIG 1-98 J Clin Oncol 2007 25 486 492 10.1200/JCO.2006.08.8617 17200148

[B5] Coombes RC Kilburn LS Snowdon CF Paridaens R Coleman RE Jones SE Jassem J Velde CJH Delozier T Alvarez I Mastro L Ortmann O Diedrich K Coates AS Bajetta E Holmberg SB Dodwell D Mickiewicz E Andersen J Lønning PE Cocconi G Forbes J Castiglione M Stuart N Stewart A Fallowfield LJ Bertelli G Hall E Bogle RG Carpentieri M Colajori E Subar M Ireland E Bliss JM on behalf of the Intergroup Exemestane Study Survival and safety of exemestane versus tamoxifen after 2-3 years' tamoxifen treatment (Intergroup exemestane study): a randomized controlled trial Lancet 2007 369 559 570 10.1016/S0140-6736(07)60200-1 17307102

[B6] Jakesz R Jonat W Gnant M Mittlboeck M Greil R Tausch C Hilfrich J Kwasny W Menzel C Samonigg H Seifert M Gademann G Kaufmann M on behalf of the ABCSG and the GABG Switching of postmenopausal women with endocrine-responsive early breast cancer to anastrozole after 2 years' adjuvant tamoxifen: combined results of ABCSG trial 8 and ARNO 95 trial Lancet 2005 366 455 462 10.1016/S0140-6736(05)67059-6 16084253

[B7] Goss PE Ingle JN Martino S Robert NJ Muss HB Piccart MJ Castiglione M Tu D Shepherd LE Pritchard KI Livingston RB Davidson NE Norton L Perez EA Abrams JS Cameron DA Palmer MJ Pater JL Randomized trial of letrozole following tamoxifen as extended adjuvant therapy in receptor-positive breast cancer: updated findings from NCIC CTG MA. 17 J Natl Cancer Inst 2006 98 1162 16145047 10.1093/jnci/dji250

[B8] Jakesz R Greil R Gnant M Schmid M Kwasny W Kubista E Mlineritsch B Tausch C Stierer M Hofbauer F Renner K Dadak C Rücklinger E Samonigg H Austrian Breast and Colorectal Cancer Study Group Extended adjuvant therapy with anastrozole among postmenopausal breast cancer patients: results from the randomized Austrian Breast and colorectal Cancer Study Group Trial 6a J Natl Cancer Inst 2007 99 1845 1853 10.1093/jnci/djm246 18073378

[B9] Smith IE Dowsett M Yap YS Walsh G Lønning PE Santen RJ Hayes D Adjuvant aromatase inhibitors for early breast cancer after chemotherapy-induced amenorrhoea: caution and suggested guidelines J Clin Oncol 2006 24 2444 2447 10.1200/JCO.2005.05.3694 16735701

[B10] Burstein HJ Mayer E Patridge AH O'Kane H Litsas G Come SE Hudis CA Goldstein DF Muss HB Winter EP Garber JE Inadvertent use of aromatase inhibitors in patients with breast cancer with residual ovarian function: cases and lessons Clin Breast Cancer 2006 7 158 161 10.3816/CBC.2006.n.026 16800976

[B11] Hargis JB Nakajima ST Resumption of menses with initiation of letrozole after five years of amenorrhea on tamoxifen: caution needed when using tamoxifen followed by aromatase inhibitors Cancer Invest 2006 24 174 177 10.1080/07357900500524538 16537187

[B12] Sasa M Bando Y Takahashi M Hirose T Nagao T Screening for basal marker expression is necessary for detection of therapeutic strategy for triple-negative breast cancer J Surg Oncol 2008 97 30 34 10.1002/jso.20906 17929254

[B13] Nomura Y Koyama H Ohashi Y Watanabe H Arimidex Clinical Study Committee linical Dosage Determination of a New Aromatase Inhibitor, Anastrozole, in Postmenopausal Japanese Women with Advanced Breast Cancer Clin Drug Invest 2000 20 357 369 10.2165/00044011-200020050-00007

[B14] Iveson TJ Smith IE Ahern J Smithers DA Trunet PF Dowsett M Phase I study of the oral nonsteroidal aromatase inhibitor CGS 20267 in postmenopausal patients with advanced breast cancer Cancer Res 1993 53 266 270 8417819

[B15] Bajetta E Zilembo N Dowsett M Guillevin L Di Leo A Celio L Martinetti A Marchianò A Pozzi P Stani S Bichisao E Double-blind, randomised, multicentre endocrine trial comparing two letrozole doses, in postmenopausal breast cancer patients Eur J Cancer 1999 35 208 213 10.1016/S0959-8049(98)00392-X 10448261

[B16] Geisler J Haynes B Anker G Dowsett M Lønning PE Influence of letrozole and anastrozole on total body aromatization and plasma estrogen levels in postmenopausal breast cancer patients evaluated in a randomized, cross-over study J Clin Oncol 2002 20 751 757 10.1200/JCO.20.3.751 11821457

[B17] Geisler J Detre S Berntsen H Ottestad L Lindtjørn B Dowsett M Einstein Lønning P Influence of neoadjuvant anastrozole (Arimidex) on intratumoral estrogen levels and proliferation markers in patients with locally advanced breast cancer Clin Cancer Res 2001 7 1230 1236 11350888

[B18] Chen C Sakoda LC Doherty JA Loomis MM Fish S Ray RM Lin MG Fan W Zhao LP Gao DL Staisberg H Feng Z Thomas DB Genetic variation in CYP19A1 and risk of breast cancer and fibrocystic breast conditions among women in Shanghai, China Cancer Epidemiol Biomarkers Prev 2008 17 3457 3466 10.1158/1055-9965.EPI-08-0517 19064562 PMC2760732

[B19] Zhang L Gu L Qian B Hao X Zhang W Wei Q Chen K Association of genetic polymorphisms of ER-alpha and the estradiol-synthesizing enzyme CYP17 and CYP19 with breast cancer risk in Chinese women Breast Cancer Res Treat 2009 114 327 338 10.1007/s10549-008-9998-0 18629629

